# Identification of *NADPH Oxidase* Genes Crucial for Rice Multiple Disease Resistance and Yield Traits

**DOI:** 10.1186/s12284-023-00678-5

**Published:** 2024-01-03

**Authors:** Yong Zhu, Hao Su, Xin-Xian Liu, Ji-Fen Sun, Ling Xiang, Yan-Jing Liu, Zhang-Wei Hu, Xiao-Yu Xiong, Xue-Mei Yang, Sadam Hussain Bhutto, Guo-Bang Li, Yuan-Ying Peng, He Wang, Xu Shen, Zhi-Xue Zhao, Ji-Wei Zhang, Yan-Yan Huang, Jing Fan, Wen-Ming Wang, Yan Li

**Affiliations:** State Key Laboratory of Crop Gene Exploration and Utilization in Southwest China, Sichuan Agricultural University, Chengdu, 611130 China

**Keywords:** Rice, *Rbohs*, Multiple disease resistance, *M. oryzae*, *R. solani*, *Xoo*, Yield traits

## Abstract

Reactive oxygen species (ROS) act as a group of signaling molecules in rice functioning in regulation of development and stress responses. Respiratory burst oxidase homologues (Rbohs) are key enzymes in generation of ROS. However, the role of the nine *Rboh* family members was not fully understood in rice multiple disease resistance and yield traits. In this study, we constructed mutants of each *Rboh* genes and detected their requirement in rice multiple disease resistance and yield traits. Our results revealed that mutations of five *Rboh* genes (*RbohA*, *RbohB*, *RbohE*, *RbohH*, and *RbohI*) lead to compromised rice blast disease resistance in a disease nursery and lab conditions; mutations of five *Rbohs* (*Rboh*A, *RbohB*, *RbohC*, *RbohE*, and *RbohH*) result in suppressed rice sheath blight resistance in a disease nursery and lab conditions; mutations of six *Rbohs* (*Rboh*A, *RbohB*, *RbohC*, *RbohE*, *RbohH* and *RbohI*) lead to decreased rice leaf blight resistance in a paddy yard and ROS production induced by PAMPs and pathogen. Moreover, all *Rboh* genes participate in the regulation of rice yield traits, for all *rboh* mutants display one or more compromised yield traits, such as panicle number, grain number per panicle, seed setting rate, and grain weight, resulting in reduced yield per plant except *rbohb* and *rbohf*. Our results identified the *Rboh* family members involved in the regulation of rice resistance against multiple pathogens that caused the most serious diseases worldwide and provide theoretical supporting for breeding application of these *Rbohs* to coordinate rice disease resistance and yield traits.

**Supplementary Information:**

The online version contains supplementary material available at 10.1186/s12284-023-00678-5.

## Introduction

Plants employ pathogen-associated molecular pattern (PAMP)-triggered immunity (PTI) and effector-triggered immunity (ETI) to counterattack the invasion of pathogens (Jones and Dangl 2006). The PAMPs were perceived by cell surface-localized plant pattern recognition receptors (PRRs), which subsequently activate downstream early immune responses, such as activation of mitogen-activated protein kinase (MAPK) cascade, influx of intracellular calcium, reactive oxygen species (ROS) production, and callose deposition at infected sites (Boller and Felix 2009). Pathogen-secreted effectors were directly or indirectly recognized by its cognate resistance genes, which subsequently activate strong resistance resulting in hypersensitive responses accompanying ROS production and fast cell death at the infection sites (Yuan et al. 2021; Zhou and Zhang 2020).

ROS, such as superoxide radicals (O•_2_^−^), hydroxyl radicals (OH^•^) and hydrogen peroxide (H_2_O_2_), have been identified as a group of signaling molecules in plants functioning in regulation of growth, development, and stress responses (Chapman et al. 2019; Saxena et al. 2016). Respiratory burst oxidase homologues (Rbohs), known as Nicotinamide adenine dinucleotide phosphate (NADPH) oxidases (NOXs), are key enzymes in generation of O•_2_^−^ by catalyzing the transfer of electrons from NADPH to oxygen (O_2_) in plants(Kaur et al. 2014; Suzuki et al. 2011).

NOXs were first identified in human cells for catalyzing the production of ROS (Segal and Abo 1993). In plants, Rbohs were characterized as ROS synthetic enzymes based on their sequence similarity to the phagocyte gp91^phox^/NOX2 in human (Keller et al. 1998). Rbohs contain six transmembrane domains, a N-terminal region with Ca^2+^-binding EF-hands, and a C-terminal NADPH- and flavin adenine dinucleotide (FAD)-binding cytoplasmic region (Torres et al. 2002). In the transmembrane domains, two pairs of histidine residues were located on third and fifth transmembrane domains and attached by the heme groups to facilitate electron transport (Finegold et al. 1996). N-terminus contains two calcium-binding EF hand motifs and two phosphorylation sites required for the regulation of enzyme activity (Torres et al. 2002). C-terminus-contained FAD- and NADPH-binding domains are highly conserved in NADPH family members (Kaur et al. 2018).

Arabidopsis thaliana (Arabidopsis) *Rboh* family contain 10 genes and is the best characterized *Rboh* family in plant. The 10 *Rbohs*, from *RbohA* to *RbohJ*, display diverse expression patterns and play differential roles in development and stress responses (Chapman et al. 2019). For example, *RbohE* is involved in the formation and development of pollen grains. *rbohe* mutants showed reduced ROS accumulation in the anther, leading to delayed programmed cell death of the tapetum, which impairs pollen viability (Xie et al. 2014). *RbohD* is participated in biotic and abiotic stress responses (Lee et al. 2013; Torres et al. 2002) and is phosphorylated on its N-terminal cytoplasmic region by a variety of protein kinases, including receptor-like cytoplasmic kinases (RLCKs) such as BOTRYTIS INDUCED KINASE 1 (BIK1). Upon PAMP treatment, BIK1 directly phosphorylates RbohD at N-terminal specific sites to enhance ROS generation thus improves plant immunity(Kadota et al. 2014; Li et al. 2014). Moreover, *RbohD* and *RbohF* regulate ABA- and ethylene-induced stomatal responses (Sierla et al. 2016), as well as the complicated interaction between the ABA-, ethylene-, JA- and SA-signaling pathways (Chapman et al. 2019; Desikan et al. 2006; Maruta et al. 2011).

Rice *Rboh* family contains 9 members widely participating in rice growth, development, and stress responses (Kaur et al. 2018). *OsRbohA*, *OsRbohD*, and *OsRbohE* are involved in rice tolerance against salt stress because the transcription of these genes is induced in the roots of a tolerant cultivar (Saini et al. 2018). *OsRbohB* and *OsRbohE* are participated in abscisic acid-induced H_2_O_2_ production (Ni et al. 2019). *OsRbohC* is induced by arsenic stress and possibly participates in restoring root system architecture under arsenic stress (Ghate et al. 2022). *OsRbohD* and *OsRbohF* collaborate with photoreceptor protein phytochrome B to regulate transcripts in response to light stress (Fichman et al. 2023). *OsRbohF* also improves salt tolerance in rice by boosting the accumulation of ROS, which is required for the activation of downstream stress-responsive genes and K^+^ uptake transporters (Liu et al. 2020). *OsRbohH* and *OsRbohI* are involved in rice submergence resistance and highly induced by full submergence with increased ROS accumulation, indicating the important role of the two genes in maintaining homeostasis during submergence (Wu and Yang 2016). *OsRbohH* suppresses ethylene-induced aerenchyma formation in roots by mediating ROS production. *osrbohh* mutants displayed reduced ROS accumulation and inducible aerenchyma formation in rice roots; In contrast, the expression of *OsRbohH* is induced to greatly higher levels in cortical cells under oxygen-deficient conditions (Yamauchi et al. 2017).

Except abiotic stresses, rice is also threatened by multiple disease pathogens during the whole growth period. For example, the semi-necrotrophic fungal pathogen *Magnaporthe oryzae* (*M. oryzae*) causes blast disease occurred on leaves, panicles, and grains through the whole growth period (Talbot 2003); the necrotrophic fungal pathogen *Rhizoctonia solani* (*R. solani*) causes sheath blight on sheaths and leaves (Molla et al. 2020); the bacterial pathogen *Xanthomonas oryzae* pv. *oryzae* (*Xoo*) causes bacterial leave blight (Chukwu et al. 2019). *OsRbohA* RNAi plants were more susceptible to sheath blight (Chen et al. 2023). *OsRbohB* and *OsRbohH* possibly play an important role in rice fungal disease resistance because they are the major NADPH oxidases responsive to chitin. Upon *M. oryzae* infection, *rbohb* mutants displays greater lesion length and supports more extensive fungal growth than the wild type, indicating an essential role of *OsRbohB* in rice blast disease resistance (Nagano et al. 2016). Moreover, OsRbohB interacted with receptor like kinases RLK20, RLK21, and RLK20, which inhibits its enzyme activity to negatively regulate rice resistance to *Xoo* (Mei et al. 2022).

In a previous study, we found that *OsRbohB* and *OsRbohC* (previously named as *OsRbohF*, (Jang et al. 2012) were responsive to *M. oryzae*(Li et al. 2019). A virulent strain Guy11 significantly suppressed the RNA amounts of the two genes in rice varieties Kasalath, NPB and TP309, implying a role of *OsRbohs* in rice-*M. oryzae* interaction (Li et al. 2019). However, the roles of the nine *OsRbohs* were not fully understood in rice multiple disease resistance and yield traits. In this study, we systemically explore the roles of all the nine *OsRboh* genes in the regulation of rice multiple disease resistance and yield traits. We detected the diverse expression patterns of all the nine *Rbohs* through the whole growth period and constructed the mutants of these OsRbohs. We examined the resistance of these mutants against *M. oryzae*, *R. solani*, and *Xoo*, respectively, and the production of ROS induced by PAMPs and pathogen. We also analyzed the agronomic traits of these mutants. Our results would shed light on the function of the *Rboh* family in rice, as well as provide experimental reference for breeding application of these genes to coordinate disease resistance and yield traits in rice.

## Results

### Rice *Rboh* Family Members are Differentially Responsive to PAMPs

To make out the evolutional relationship of the Rbohs between Arabidopsis and rice, we constructed a phylogenetic tree via BLAST analysis. From this phylogenetic tree, the Rboh members can be classified into three clades, of which clade I contains OsRbohB, OsRbohH, and OsRbohI belonging to the same clade close to AtRbohD; Clade II contains rice OsRbohF, OsRbohG, OsRbohA, and OsRbohC belonging to a clade close to AtRbohE and AtRbohF; Clade III contains OsRbohD and OsRbohE, and Arabidopsis AtRbohH and AtRbohJ (Fig. S1). These clades imply that rice OsRbohs may play conserved but differential functions in comparison with those of Arabidopsis in disease resistance and growth.

**Fig. 1:**
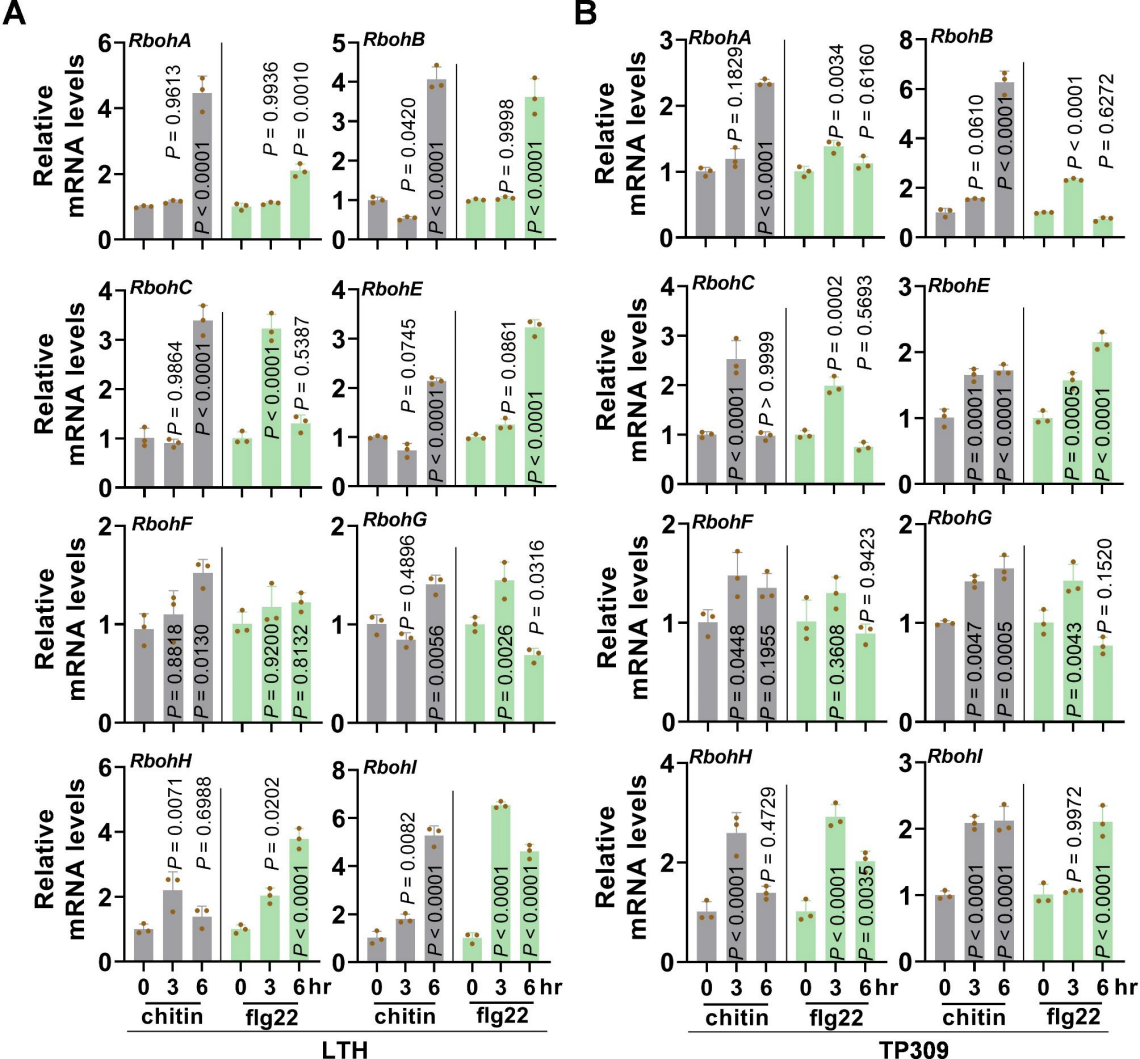
*Rbohs* are responsive to pathogen-associated molecular patterns (PAMPs). **A** and **B**, Reverse-transcription quantitative polymerase chain reaction (RT-qPCR) data show the expression patterns of *Rbohs* in leaves of a susceptible cultivar Lijiang xin Tuan Heigu (LTH, **A**) and Taipei 309 (TP309, **B**) at 3- and 6-hours post treatment of PAMPs chitin and flg22, respectively. Data are shown as mean ± SD (n = 3 independent repeats). All the *P* values were determined by One-way ANOVA analysis, and the colored dots associated with the bars indicate the value of biologically independent samples

Although some *Rbohs*, including *RbohA*, *RbohB*, *RbohC*, and *RbohH*, have been characterized as regulators in rice disease resistance against *Xoo* or *M. oryzae*, the roles of the other *Rbohs* are unknown. To systemically explore the roles of the *Rboh* family members in rice disease resistance, we first tested their response to PAMPs by examining their expression pattern upon the treatment of chitin and flg22 in Lijiang xin Tuan Heigu (LTH) and Taipei 309 (TP309). LTH is a *Japonica* accession sensitive to over 1300 regional *M. oryzae* isolates worldwide (Lin et al. 2001). TP309 is another Japonica accession susceptible to many *M. oryzae* isolates and widely used in rice transformation. Except *RbohD* whose expression was below detectable level in leaves, the expression of six *Rbohs* (*RbohA*, *RbohB*, *RbohC*, *RbohE*, *RbohH*, and *RbohI*) was induced by chitin- or flg22-treatment more than 2-fold higher than that of the untreated samples in both accessions (Fig. 1), indicating the involvement of these *Rbohs* in rice responses to PAMPs that derived from fungal and bacterial pathogens. In contrast, although the expression of *RbohF* and *RbohG* were also enhanced by chitin or flg22, the enhancement levels were less than 2-fold of the untreated samples, suggesting a minor role in rice responses to PAMPs (Fig. 1). These data imply the differential roles of these *Rboh*s in disease resistance, of which six *Rboh*s may participate in the modulation of rice disease resistance.

To further explore the roles of the *Rboh* family members in the regulation of rice disease resistance and yield traits, we generated mutants for each of the nine *Rboh*s using the CRISPR/Cas9 technology in TP309 background. We obtained two or more homozygous mutants for each *Rboh* gene except *RbohC*, which we obtained two mutants with the same mutation of 7-base deletion (Fig. S2). Except *rbohi-3*, all mutants carried one or more base deletion or insertion creating a stop codon resulting in truncated proteins or changed amino acid sequence at N-terminus (Fig. S2A-I). Intriguingly, *rbohi-3* carried a 3-base deletion leading to a serine deletion at A,a 338 site (Fig. S2I ), which was a possible key site for phosphorylation by upstream kinases.

### Five *OsRboh* Family Members Regulates Rice Blast Disease at Different Levels

We first identified the *Rbohs* participated in rice blast disease resistance by examining the responses of these *Rbohs* to a virulent *M. oryzae* strain GZ8 in a susceptible accession LTH, a susceptible accession TP309, and a resistance accession Pyricularia-Kanto51-m-Tsuyuake (IRBLkm-Ts) carrying a resistance (R) locus *Pi-km* (Tsumematsu et al. 2000). As shown in Fig. S3, eight *Rboh* genes were suppressed by *M. oryzae* at one or more time points in both LTH and IRBLKm-Ts, indicating the involvement of these *Rbohs* in blast disease resistance.

**Fig. 2:**
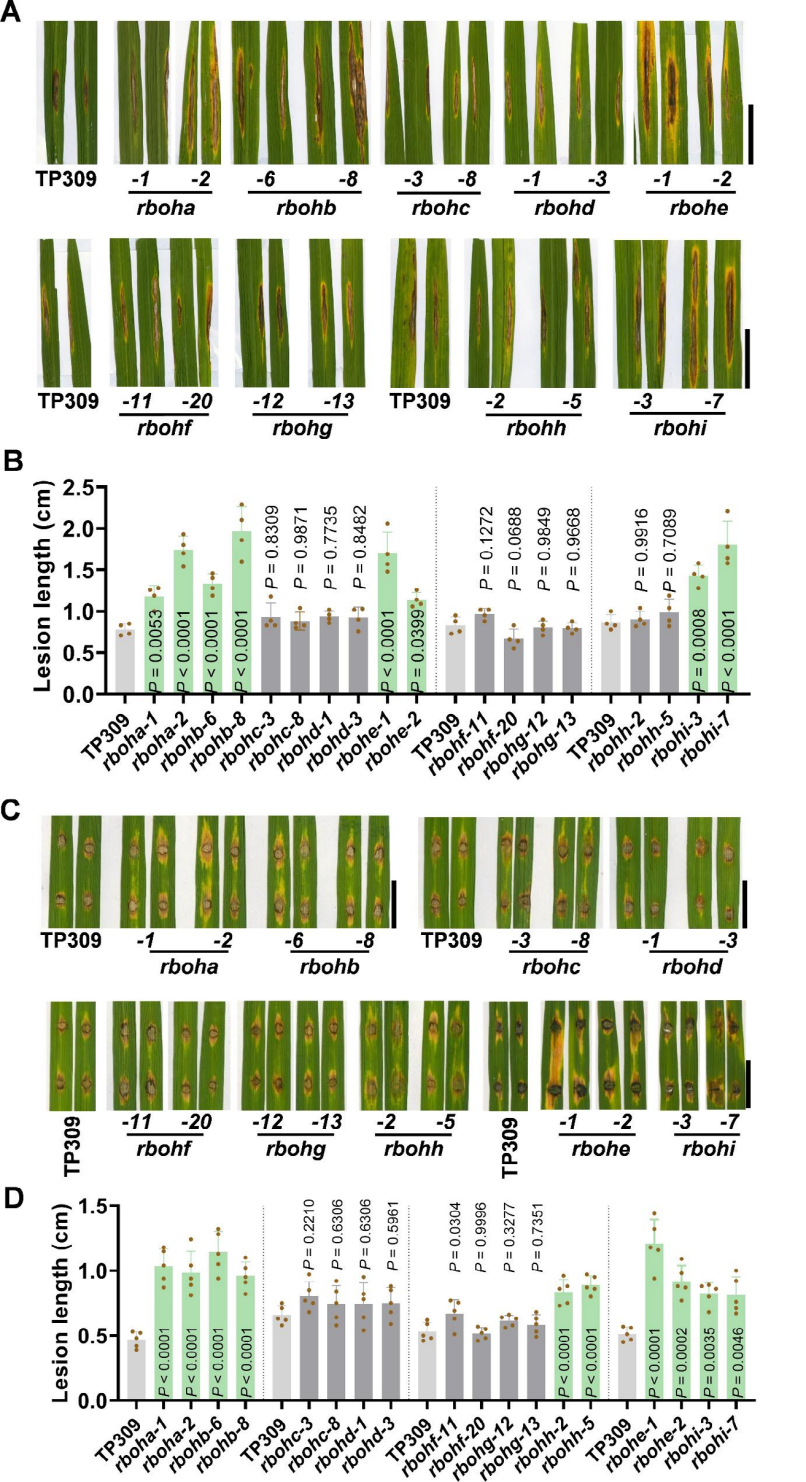
Mutations of *RbohA, RbohB, RbohE, RbohH*, and *RbohI* result in compromised blast disease resistance in rice. (**A**) Disease symptoms of *rboh* mutants and the TaPei309 (TP309) control planted in a disease nursery at tillering stage. Scale bars = 1 cm. (**B**) The lesion length of the indicated mutants in A. (**C**) Disease symptoms of *rboh* mutants and the TP309 control five days post inoculation (dpi) of *Magnaporthe oryzae* isolates GZ8 by punch-inoculation at seedling stage in lab conditions. Scale bars = 1 cm. (**D**) The lesion length of the indicated mutants in C. For B and D, the lesion length was examined by image J software. Data are shown as mean ± SD (n = 5). All the *P* values were determined by One-way ANOVA analysis, and the colored dots associated with the bars indicate the value of biologically independent samples

We then examined the blast disease resistance of these *rboh* mutants in lab conditions and in a blast disease nursery. Plants of *rboha*, *rbohb*, *rbohe*, and *rbohi* displayed exacerbated susceptibility with bigger disease lesions than the TP309 control in the disease nursery (Fig. 2A-B) and punch-inoculated with a virulent strain GZ8 in lab conditions (Fig. 2C-D), implying a key role of the four *Rbohs* in rice blast disease resistance. Moreover, *rbohh* showed exacerbated susceptibility following GZ8 punch-inoculation in lab conditions whereas a similar disease symptom like TP309 when planted in disease nursery (Fig. 2A-D), implying the involvement of *RbohH* in post-invasive defense. In contrast, *rbohc*, *rbohd*, *rbohf*, and *rbohg* displayed a compatible disease symptom and fungal growth to the TP309 control, indicating the independence of these four *Rbohs* in rice resistance against *M. oryzae*. Altogether, these results indicate that five *Rboh*s (*RbohA*, *RbohB*, *RbohE*, *RbohH*, and *RbohI*) participated in and positively modulate rice blast disease resistance.

### Five *OsRboh* Family Members Modulate Rice Sheath Blight Resistance

**Fig. 3:**
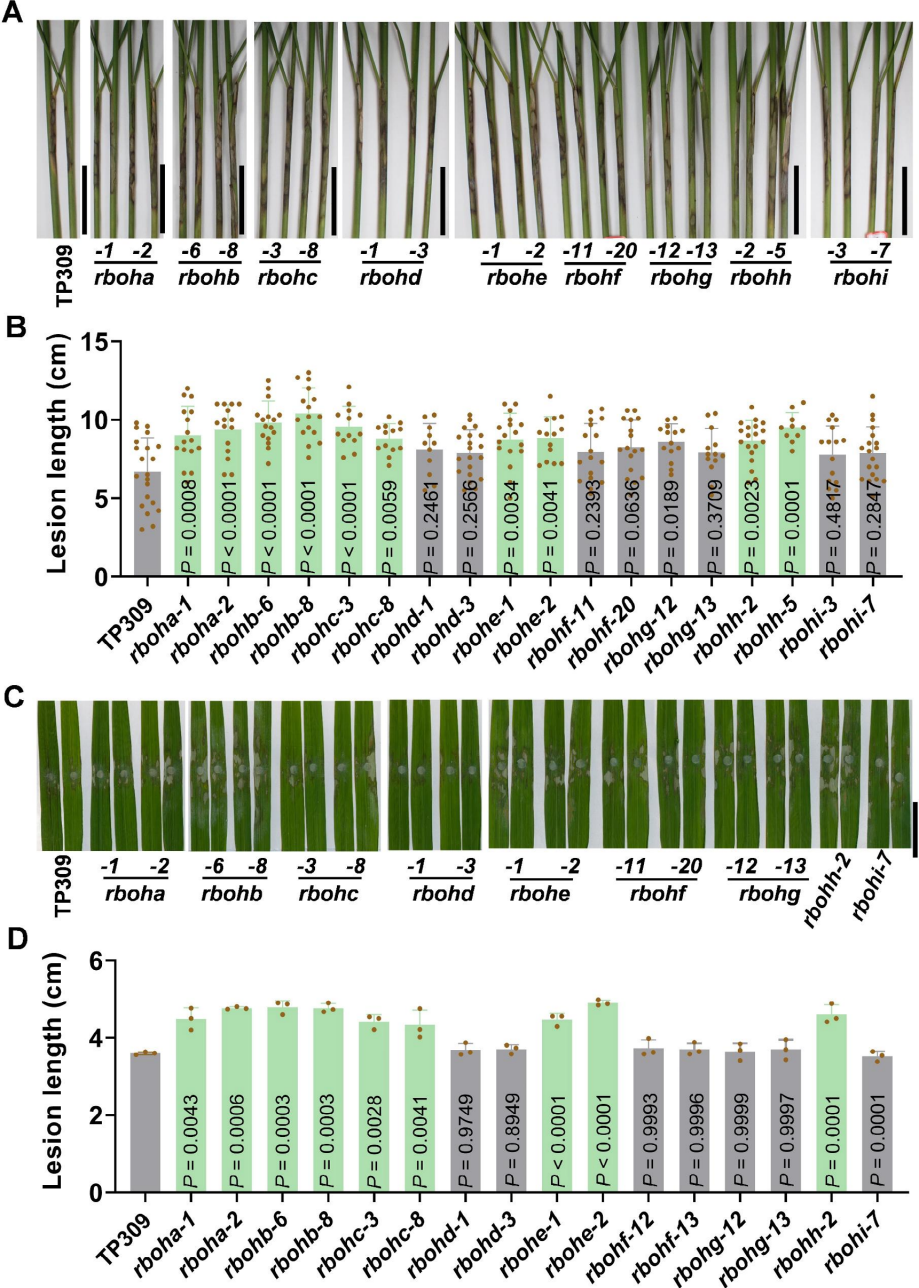
Mutations of *RbohA, RbohB, RbohC, RbohE*, and *RbohH* lead to compromised sheath blight disease resistance in rice. (**A**) Disease symptoms of the of *rboh* mutants and the TaPei309 (TP309) control planted in a disease nursery at heading stage. Scale bars = 1 cm. (**B**) Quantitative lesion length measured as in (**A**). Data are shown as mean ± SD (n > 10). Scale bars = 5 cm. (**C**) Disease symptoms of the leaves of *rboh* mutants and the TP309 control 3 days post inoculation (dpi) of *Rhizoctonia solani* isolate AG-1-IA at tillering stage. Scale bars = 2 cm. (**D**) Quantitative lesion length measured as in (**C**). Data are shown as mean ± SD (n = 3). For B and D, all the *P* values were determined by One-way ANOVA analysis, and the colored dots associated with the bars indicate the value of biologically independent samples

We next detected the resistance of these mutants to another fungal pathogen, *R. solani*, the causative agent of rice sheath blight. When planted in a disease nursery, *rboha*, *rbohb*, *rbohc*, *rbohe*, and *rbohh* showed bigger and remarkably longer disease lesions than that of the TP309 control (Fig. 3A-B). Consistently, similar results were obtained when these mutants were inoculated with a virulent *R. solani* strain AG-1-IA in lab conditions (Fig. 3C-D). These results indicate that the five *Rboh*s were involved in and positively regulated rice sheath blight resistance. In contrast, *rbohd*, *rbohf*, *rbohg*, and *rbohi* displayed disease lesions like that of TP309 (Fig. 3A-D), suggesting that these four *Rbohs* possibly not participated in rice disease resistance against *R. solani*.

### Six *OsRboh* Family Members Regulate Rice leaf Blight Resistance

**Fig. 4:**
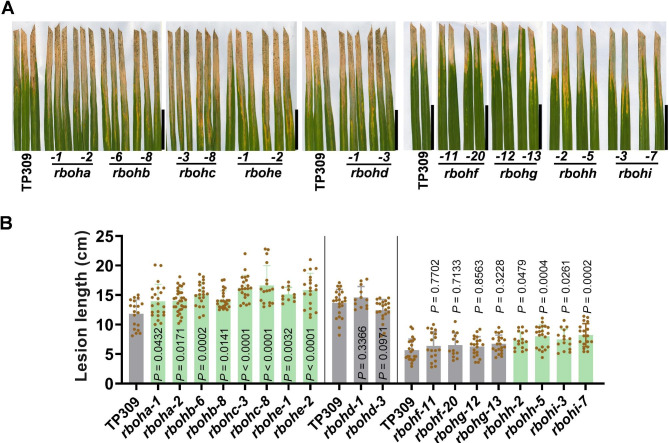
Mutations of *RbohA, RbohB, RbohC, RbohE, RbohH*, and *RbohI* lead to compromised leaf blight disease resistance in rice. (**A**) Disease symptoms of the *rboh* mutants and the TaPei309 (TP309) control 14 days post-inoculation of *Xanthomonas oryzae* pv. *oryzae* (*Xoo*) isolate PXO99 in a paddy yard at heading stage. Scale bars = 5 cm. (**B**) Quantitative lesion lengths measured as in (**A**). Data are shown as mean ± SD (n > 11). All the *P* values were determined by One-way ANOVA analysis, and the colored dots associated with the bars indicate the value of biologically independent samples

We also detected the resistance of these mutants to the bacterial pathogen, *Xoo*, which caused one of the most serious rice bacterial diseases, leaf blight disease. When inoculated with a virulent strain PXO99 in a paddy yard, *rboha*, *rbohb*, *rbohc*, *rbohe*, *rbohh*, and *rbohi* lines showed significantly longer disease lesions than that of the TP309 control, whereas *rbohd*, *rbohf*, and *rbohg* lines exhibited indistinguishable length of disease lesions (Fig. 4A-B). These results indicate that *RbohA*, *RbohB*, *RbohC*, *RbohE*, *RbohH*, and *RbohI* are required for rice leaf blight resistance.

### Six Rboh Family Members Regulate ROS Production

Based on above disease analysis, we deduced that six Rbohs, including *RbohA*, *RbohB*, *RbohC*, *RbohE*, RbohH, and *RbohI*, participate and positively regulate rice disease resistance against one or more pathogens. These results are consistent with the induction of the six *Rbohs* by PAMPs. As Rbohs functioned as enzymes to catalyze the generation of O•_2_^−^, a key ROS molecule, we then examined the early ROS production triggered by PAMPs and the late O•_2_^−^ and H_2_O_2_ accumulation induced by *M. oryzae* in these mutants.

**Fig. 5:**
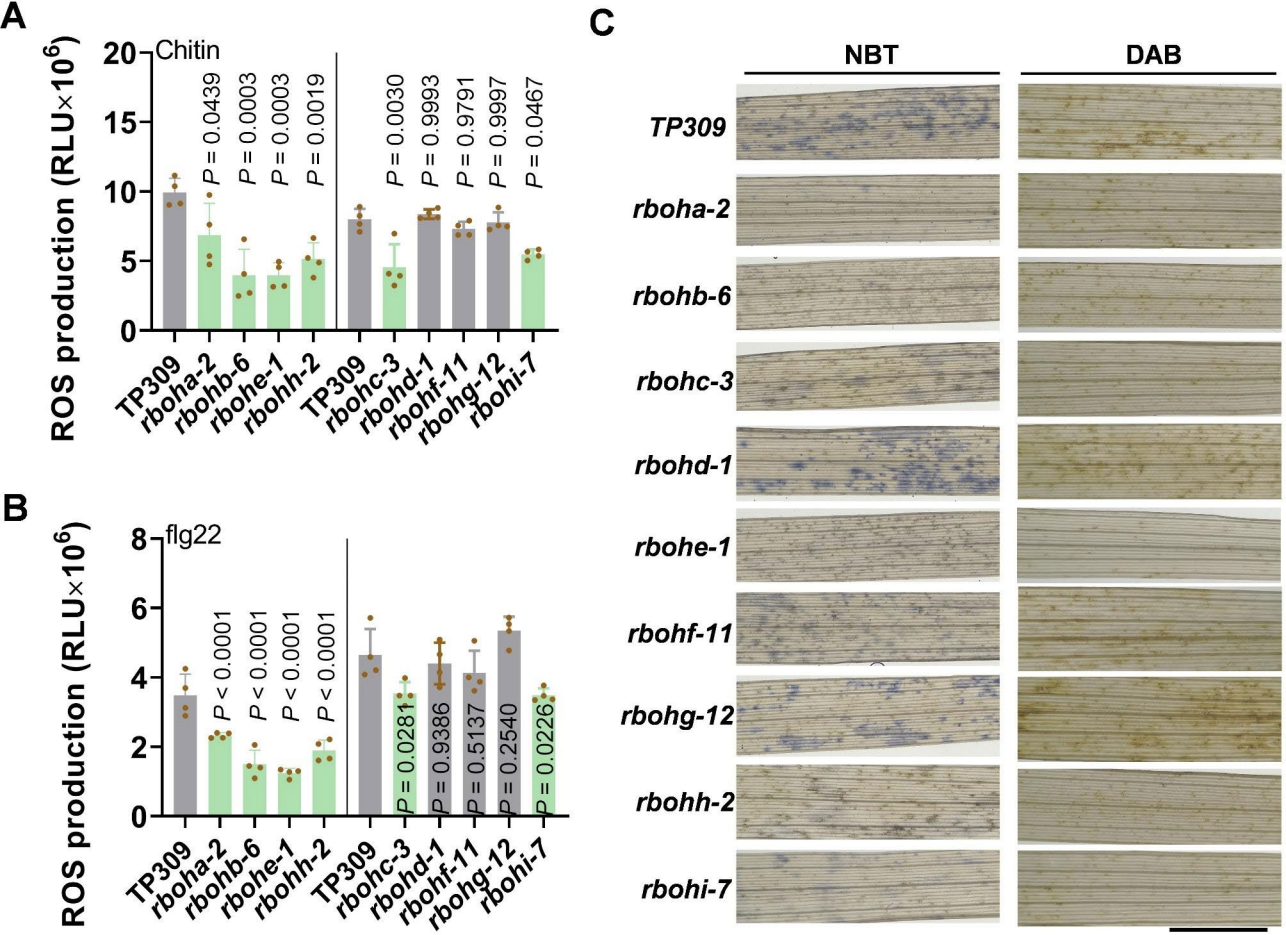
Mutations of *RbohA, RbohB, RbohC, RbohE, RbohH*, and *RbohI* suppress rice ROS production. (**A**, **B**) chitin- and flg22-induced reactive oxygen species (ROS) bursts in the leaves of the *Rboh* mutant lines and the TaPei309 (TP309) control. Data are shown as mean ± SD (n = 4). All the *P* values were determined by One-way ANOVA analysis, and the colored dots associated with the bars indicate the value of biologically independent samples. (**C**) O•_2_^−^ and H_2_O_2_ accumulation in the indicated mutants and the TP309 control at 48 h post-inoculation with *M. oryzae*. Nitrotetrazolium Blue chloride (NBT) was used as an indicator of O•_2_^−^ amounts (blue). 3, 3 N-diaminobezidin (DAB) was used as an indicator of H_2_O_2_ amounts (reddish-brown). Scale bars = 5 mm

Consistent with the disease phenotypes in these mutants, both chitin- and flg22-triggered ROS production was significantly less in *rboha*, *rbohb*, *rbohc*, *rbohe*, *rbohh*, and *rbohi* than that in the TP309 control (Fig. 5A-B). Similarly, *M. oryzae*-induced accumulation of O•_2_^−^ and H_2_O_2_ was also remarkedly compromised in these mutants compared to that in TP309 control (Fig. 5C). These results implied that these six *Rbohs* play roles in rice disease resistance by boosting ROS production. In contrast, *rbohd*, *rbohf*, and *rbohg* showed a similar ROS burst and O•_2_^−^ and H_2_O_2_ accumulation like the TP309 control (Fig. 5A-C), suggesting that the three *Rbohs* were not involved in the regulation of rice leaf disease resistance.

### Mutation of *Rboh* Family Members Lead to Diverse Changes of Yield Traits

**Fig. 6:**
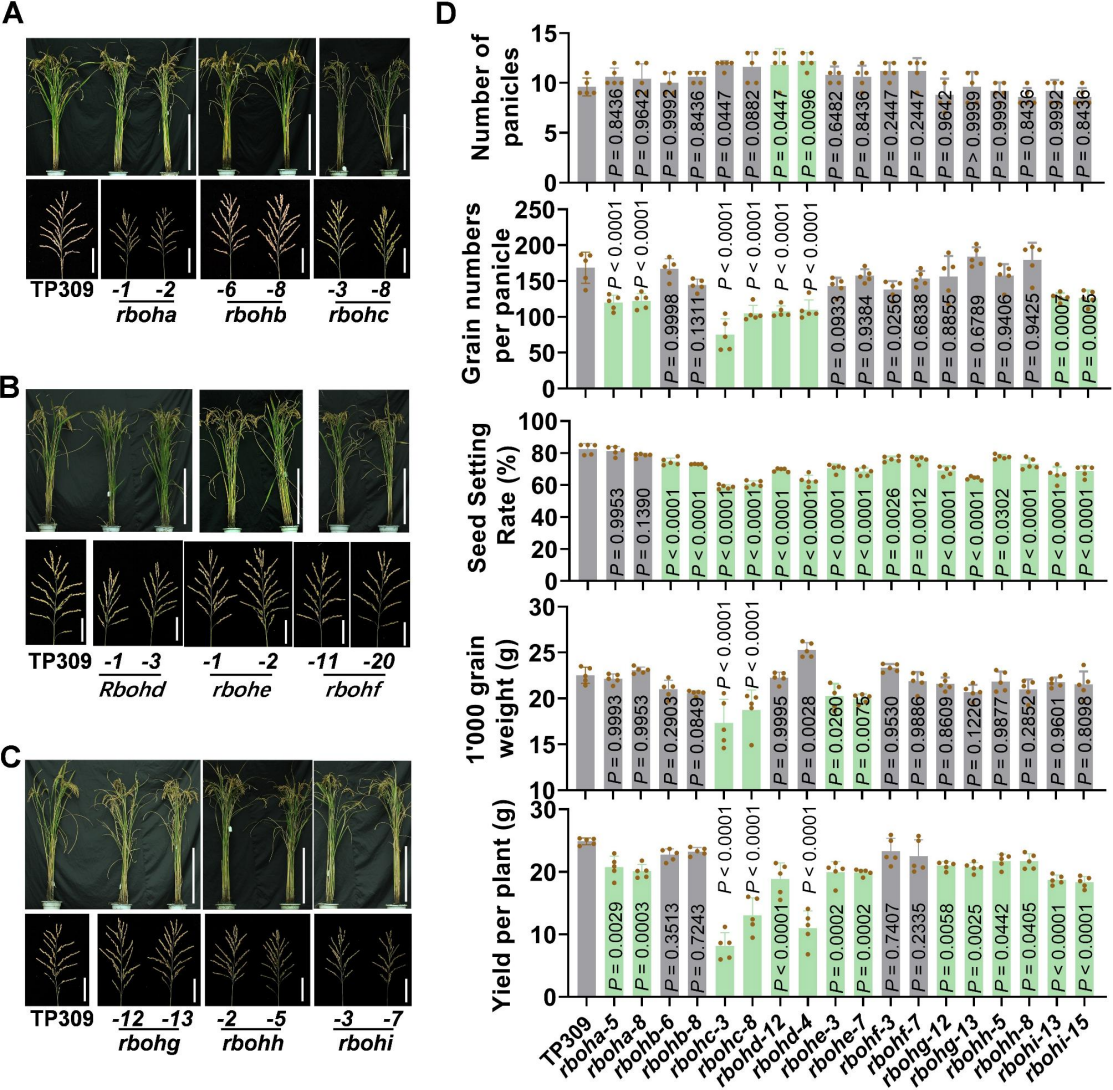
Mutations of *Rbohs* compromise rice yield traits. (**A**–**C**), The gross morphology and panicle morphology of the *Rboh* mutants and the Tapei309 (TP309) controls at the maturation stage. Scale bars, 50 cm for plants and 5 cm for panicles. (**D**) Panicle number, grain number per panicle, seed setting rates, grain weight, and yield per plant of the indicated lines planted in a paddy yard in the rice-growing season in Chengdu, Sichuan Province, Southwest of China. Data are shown as mean ± SD (n = 5 independent plants). All the *P* values were determined by One-way ANOVA analysis, and the colored dots associated with the bars indicate the value of biologically independent samples

*Rbohs* not only modulate rice resistance against multiple pathogens, but also control rice agronomic traits. We analyzed the yield traits of these *rboh* mutants that planted in a field during the rice growth season from middle April to middle September in 2022 and 2023 at Chengdu city, Sichuan Province, the southwest of China. We found that although all the mutants displayed a similar gross morphology like the TP309 control (Fig. 6A-C), they all displayed one or more defects in yield traits (Fig. 6D, Table S2). Plants of *rboha* mutants showed smaller panicles and significantly reduced grain numbers per panicles; *rbohb* lines displayed decreased seed setting rates but slightly increased panicle numbers; *rbohc* exhibited decreased plant height, significantly reduced grain numbers per panicle, seed setting rates, and grain weight; *rbohd* showed significantly increased panicle number but smaller panicles with significantly decreased grain numbers and seed setting rates; *rbohe* exhibited remarkably decreased seed setting rates and grain weight; *rbohf* displayed decreased seed setting rates whereas slightly increased panicle numbers, *rbohg* and *rbohh* showed decreased seed setting rates; *rbohi* displayed decreased plant height, smaller panicles with decreased grain numbers per panicle and seed setting rates (Fig. 6A-D, Table S2). As a result, *rboha*, *rbohc*, *rbohd*, *rbohe*, *rbohg*, *rbohh*, and *rbohi* exhibited significantly decreased grain yield, especially *rbohc* and *rbohd* lines, which showed the most reduction of yield (Fig. 6D, Table S2), indicating the requirement of the seven *Rbohs* in the development of panicle and the formation of grain yield. In contrast, although exhibited reduced seed setting rates, *rbohb* and *rbohf* showed slight but not significant yield reduction compared to the TP309 control, suggesting that these two *Rbohs* slightly affected rice yield.

### *Rboh* Family Members Show Diverse Spatio-Temporal Expression Patterns During Growth Period

As the function of *Rbohs* is associated with their expression levels, we then detect their spatio-temporal expression patterns by performing a time-course experiment to examine the relative mRNA levels (compared to that of *Ubiquitin*) of all the nine *Rbohs* in rice leaves at vegetative stage and in panicles at the reproductive stage. Based on their relative mRNA amounts, we classified the nine *Rbohs* into three groups at vegetative and panicle stages, respectively.

**Fig. 7:**
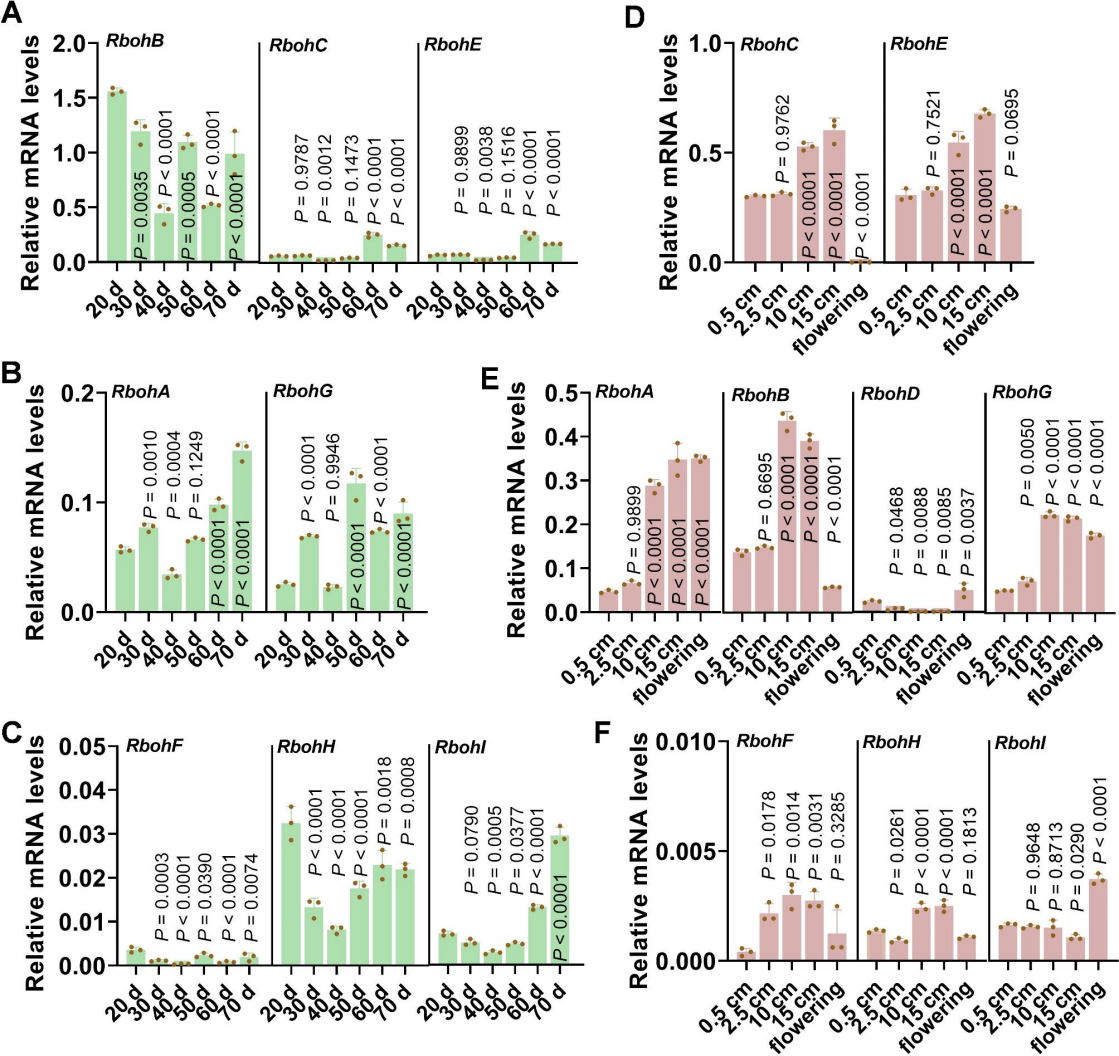
*Rboh* family members displays diverse spatio-temporal expression patterns during the rice-growing period. (**A**–**F**) Reverse-transcription quantitative polymerase chain reaction (RT-qPCR) data show the expression patterns of *Rbohs* in leaves of the TaPei309 (TP309) plants during the vegetative stage (**A**–**C**) and in panicles during the reproductive stage (**D**–**F**). Data are shown as mean ± SD (n = 3). All the *P* values were determined by One-way ANOVA analysis, and the colored dots associated with the bars indicate the value of biologically independent samples

The group 1 members at vegetative stage showed relatively high expression levels, including *RbohB*, *RbohC*, and *RbohE*. *RbohB* displayed the highest RNA amounts in leaves among the three members, whereas *RbohC* and *RbohE* showed gradually increased RNA amounts in leaves at late vegetative stage (Fig. 7A), which were consistent with the key role of *RbohB* and *RbohE* in rice disease resistance. The members in group 2 exhibited lower RNA levels compared to those in group 1, including *RbohA* and *RbohG*, which were increased gradually in leaves (Fig. 7B). The members in group 3 exhibited the lowest RNA levels, including *RbohF*, *RbohH*, and *RbohI*. *RbohH* were decreased from 30 day whereas *RbohI* was decreased from 20 to 40 day and then increased gradually; *RbohF* was low-expressed during the whole growth period (Fig. 7C). *RbohD* was below to the detectable levels. The low expression of *RbohD* and *RbohF* partially explained their absence in rice immunity in leaves.

Similarly, we also classified the nine *Rbohs* into three groups at panicle stages based their expression levels. The group 1 members exhibited the highest expression levels, including *RbohC* and *RbohE* (Fig. 7D). The group 2 exhibited relatively lower RNA levels, including *RbohA*, *RbohB*, *RbohD*, and *RbohG* (Fig. 7E). The members in group 3 exhibited the lowest RNA levels and included *RbohF*, *RbohH*, and *RbohI* (Fig. 7F). Intriguingly, the expression of *RhobA*, *RbohC*, *RbohE*, and *RbohG* was gradually enhanced during panicle development, and the expression of *RbohD* and *RbohI* was enhanced during flowering (Fig. 7D-F). These results were consistent with the defects of yield traits of these *rboh* mutants (Fig. 6), implying a positive role of these genes in the development of panicle and flower organs.

## Discussion

**Fig. 8:**
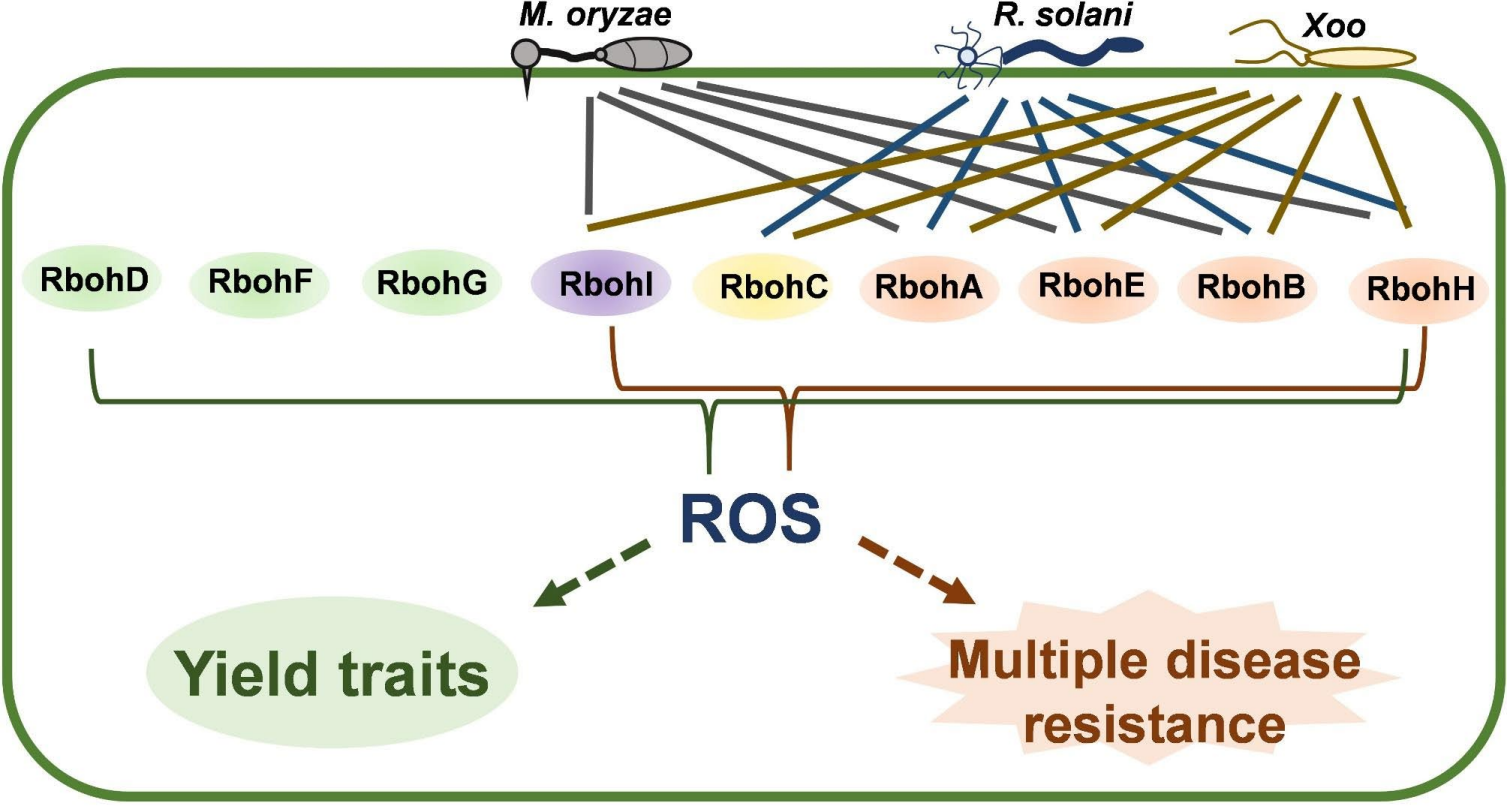
A work model depicting the differential roles of *Rbohs* in rice multiple disease resistance and growth. All the nine Rboh family members participated in the regulation of rice growth and development, and each *Rboh* preferred to differential agronomical traits. Pathogen-derived PAMPs (such as chitin and flg22) induced the expression of six *RBOHs*, including *RbohA*, *RbohB*, *RbohC*, *RbohE*, *RbohH*, and *RbohI*, which subsequently triggered the ROS production. On the one side, the enhanced accumulation of ROS improves rice resistance against both fungal and bacterial pathogens. In the other side, the enhanced ROS accumulation impact the development of panicles and grains leading to compromised yield traits and reduced yield. As a results, all the nine *Rbohs* participated in the coordination of rice yield and multiple disease resistance

In this study, we systemically explored the functions of the *Rboh* family members in the regulation of multiple disease resistance and yield traits in rice (Fig. 8; Table S2). Our results demonstrated that six *Rboh* family members, *RbohA*, *RbohB*, *RbohC*, *RbohE*, *RbohH*, and *RbohI*, modulate rice leaf disease resistance. *RbohA*, *RbohB*, *RbohE* and *RbohH* are involved in multiple disease resistance against fungal pathogen *M. oryzae*, *R. solani*, and bacterial pathogen *Xoo*; *RbohI* modulates resistance against *M. oryzae* and *Xoo*, *RbohC* participates in the regulation of resistance against *R. solani* and *Xoo*. Moreover, all *Rboh* family members regulate rice yield traits, such as panicle number, grain number per panicle, seed setting rate, and grain weight, and mutations of seven *Rbohs* result in reduced yield per plant associated with one or more compromised yield traits (Fig. 6).

Intriguingly, although it is possible that these *Rbohs* play a redundant role each other, mutations in a single *Rboh* gene in this study led to compromised resistance against one or more pathogens or defected yield traits, implying essential function of each *Rboh* that cannot be replaced by any other *Rboh* in rice development or disease resistance. However, the preference and effect of these *Rbohs* on different disease resistance and yield traits are differential from each other. For example, compared to the TP309 control and other mutants, *rbohb*, *rbohe*, and *rbohi* showed the biggest lesions and supported the most fungal growth when inoculated with *M. oryzae* or planted in a blast nursery, indicating the key role of *RbohB*, *RbohE*, and *RbohI* in rice blast disease resistance. In contrast, *rbohb* displayed the most significant susceptibility to *R. sonalo*, whereas *Rbohc* and *Rbohe* exhibited the longest disease lesions following *Xoo* inoculation. *RbohC* and *RbohD* played crucial roles in the regulation of yield traits, for *rbohc* and *rbohd* displayed the most reduction of yield. Moreover, although *RbohF* and *RbohG* was expressed in both leaves and panicles, they were specifically functioned in panicles to regulate seed setting rate and had no effect on leaf disease resistance. Altogether, our results revealed the diverse preference of *Rbohs* in the regulation of rice different disease resistance and yield traits.

The spatio-temporal expression patterns of *Rbohs* might be critical in determining their multiplicity of functions. For example, in 4-week-old Arabidopsis plants, *AtRbohF* was mainly expressed in the vascular tissue, while *AtRbohD* was expressed in disperse areas of the leaf lamina and stomatal cells, which was consistent with the higher ROS production in *AtRbohD*-overexpressed plants than that in *AtRbohF*-overexpressed plants triggered by PAMPs or pathogens (Morales et al. 2016). In this study, compared to the other *Rbohs* in rice, *RbohB* showed the most mRNA amounts in rice leaves, which was consistent with its key role in the regulation of leave disease resistance and defense responses (Figs. 2, 3, 4 and 5). Conversely, *RbohD* was low-expressed in leaves but expressed higher in panicles during flowering (Fig. 7), which was consistent with the lowest seed setting rate of *rbohd* in all tested *rboh* mutants (Fig. 6), indicating a key role of *RbohD* in the development of flowering organs.

Except for the spatio-temporal expression diversity, the preferences of *Rbohs* were probably caused by the different regulatory factors or hormone signalings functioning upstream or downstream. For example, RbohB protein was localized in microdomains and interacted with Rac1, a Rac/ROP small G protein acting as a molecular switch in defense signaling (Nagano et al. 2016). Disruption of the OsRac1–OsRbohB interaction resulted in compromised ROS production (Kosami et al. 2014). Whether other resistance-related Rboh family members also localized in microdomains and interacted with Rac1 to improve ROS production was unknown and need further study.

It was intriguingly that the mRNA amounts of most *Rbohs* were suppressed by *M. oryzae* in both susceptible and resistance varieties (Fig. S3). These results seemed be conflictive to their positive function in ROS production and indicated the existence of other regulation mechanism on translational level or enzyme activates to control the functions of *Rbohs*. It was reported that upon *M. oryzae* inoculation, the N terminus of RbohB was bound and phosphorylated by a receptor-like cytoplasmic kinase (RLCK)118, which was phosphorylated by another receptor like kinase SPL11 CELL-DEATH SUPPRESSOR 2 (SDS2) (Fan et al. 2018). However, the phosphor-sites specifically modified by OsRLCK118 in RbohB need further investigation. A recent study demonstrated that RbohB is the main NADPH oxidase controlling ABA-induced H_2_O_2_ production and related physiological responses in rice. DMI3, a Ca^2+^/calmodulin-dependent protein kinase acting as an important positive regulator of abscisic acid (ABA) signaling, directly interacts with and activates RbohB by phosphorylating RbohB at Ser-191 (Wang et al. 2023). The activated RbohB subsequently catalyzes the production of H_2_O_2_, which is in turn required for ABA-induced activation of OsDMI3 (Wang et al. 2023). Based on these results, we predicted that although the mRNA amounts of *RbohB* and other *Rbohs* were suppressed by *M. oryzae*, the phosphorylation of Rbohs by kinases at specific Ser/Thr sites was a crucial modification that required for the enzyme activation of Rboh proteins. However, whether the other Rbohs except for RbohB were phosphorylated by RLCK118 or OsDMI3 needs further investigation.

## Conclusions

In this study, we functionally characterized rice *Rbohs* in resistance against the most serious disease worldwide and agronomic traits. Our data demonstrated that six *Rbohs* participated in rice multiple disease resistance and ROS production induced by PAMPs and pathogen; all of the nine *Rbohs* were involved in the regulation of yield traits and mutations of seven *Rbohs* led to compromised grain yield per plant. These results revealed the function of the *Rbohs* family in rice disease resistance and yield, as well as provided experimental reference for breeding application of these genes to coordinate disease resistance and yield traits in rice.

## Materials and Methods

### Plant Materials and Growth Conditions

Rice (*Oryza sativa*) *japonica* cultivars Taipei309 (TP309), a susceptible line Lijiang xin Tuan Heigu (LTH), and its resistant monogenic line IRBLkm-Ts were used in this study. For disease resistance analysis and yield trait analysis, rice plants were grown in a disease nursery and a paddy yard in Wenjiang, Chengdu, Southwest of China in the normal rice growing season from mid-April to mid-September, respectively. For resistance response analysis, rice plants were grown in a greenhouse (28 ± 2 ℃, 70–75% relative humidity) under 12-h/12-h light/dark cycles.

### CRISPR/Cas9 Plasmid Construction and Mutant Screen

The CRISPR (clustered regularly interspaced short palindromic repeats)/Cas9 plasmids were constructed as previously reported (Xie et al. 2015). The guide RNA (gRNA) sequences were screened by the Cas-OFF inder system (http://www.rgenome.net/cas-offinder/) to avoid potential off-target sites with the screen parameters to allow less than three base-pair mismatches and one DNA/RNA bulge. In brief, to construct the plasmids, the gRNA primers F and R were annealed to generate the gRNA fragment containing sticky ends for cloning. Then the gRNA fragment was inserted into the clone site of the pRGEB32 vector with T4 DNA ligase (NEB). The vector was transformed into *Agrobacterium* strain *EHA105* for rice transformation. To identify the mutation sites of these mutants, the genomic DNA of transgenic plants was extracted and amplified using the primers flanking the designed target site (Table S3). The PCR products (300–500 bp) were sequenced and aligned with the wild type genome sequence to identify the mutation sites.

### Reverse-Transcription Quantitative Polymerase Chain Reaction (RT-qPCR)

Rice leaves or panicles at indicated growth stage, or the leaves inoculated with or without *M. oryzae* were collected for RNA extraction. We extracted the total RNA using the TRIzol reagent (Invitrogen) and conducted the reverse transcription with 1 µg RNA to obtained cDNA using the PrimeScript™ RT reagent Kit with gDNA Eraser following the manufacturer’s instruction (TaKaRa Biotechnology, Dalian, China). We performed RT-qPCR using SYBR Green mix (QuantiNova SYBR Green PCR Kit, Qiagen) with the indicated primers (Table S3). To normalize the mRNA amounts of the detected genes, rice *Ubiquitin-1* (*UBQ*) gene was selected as the internal reference. Data were analyzed with the SPSS software using a one-way ANOVA followed by Tukey analysis or Student’s *t-*test with significant differences.

### Pathogen Infection and Microscopic Observation

The disease phenotype of blast disease and sheath blight disease were observed in a disease nursery. Blast disease were observed at tillering stage and the fungal lesion length was measured. Sheath blight disease were observed at heading stage and the lesion length on the sheathes was measured for statistics analysis.

The *M. oryzae* virulent isolate GZ8 were used to analyze rice blast disease resistance in lab conditions. GZ8 were cultivated in petri dish with rice flour medium (20 g L^− 1^ flour, 2 g L^− 1^ yeast extract, 15 g L^− 1^ agar) at 28 ℃ with 12-h/12-h light/ dark cycles. Seven days later, the hyphae growing on the medium were scraped off for sporulation. After 2 days, the spores were collected for punch-inoculation (1 × 10^5^ spores/ml) or spray-inoculation (5 × 10^5^ spores/ml). Disease lesions were photographed five days post-inoculation (dpi) and the lesion length was measured by image J software.

The *R. solani* isolate AG-1-IA was used to assess rice sheath blight disease resistance in lab conditions. AG-1-IA was incubated in petri dish with PDA medium for 2 days and cut into 5 mm-diameter circles and inoculated on the leaves at tillering stage. In brief, small lumps of mycelium were placed in the middle of the leaves at 26 ℃ with high-humidity conditions for 2 days. Lesion length on the inoculated leaves were measured for statistics analysis.

The *Xoo* isolate PXO99 was used to test rice leaf bright disease resistance in a paddy yard. PXO99 was incubated with TSA medium for 1 days and suspended in sterile water for inoculation at concentration O.D._600_ = 1. Leaves at tillering stage were inoculated with PXO99 via cutting at leaf tips by scissors covered with bacteria solution. After 14 days, all inoculated leaves were collected for lesion measurement and analysis.

### ROS Burst Assay

We examined the production of ROS triggered by PAMPs flg22 and chitin, respectively. the leaves of the indicated mutant lines were cut into 3 × 3 mm^2^ squares with six scrapes and incubated in 200 µl ddH_2_O in a 96-well plate overnight to eliminate basal ROS elicited by damage. Then, the strips were treated with 20 µg/ml chitin in 200 µl reaction buffer (20 mM luminol, 10 µg/ml horseradish peroxidase (Sigma-Aldrich Shanghai Trading Co Ltd, Shanghai, China)). Data were collected as relative luminescence units using a GloMax96 Microplate Luminometer (Promega Biotech Co., Ltd, Beijing, China) for more than 30 min.

### O•_2_^−^ and H_2_O_2_ Staining

We collected leaves of three-leaf stage plants 48 h post spray-inoculation with *M. oryzae* for O·_2_^−^ and H_2_O_2_ staining. O·_2_^−^ histochemical staining was performed using NBT (Nitrotetrazolium Blue chloride) following a previous report (Wu et al. 2017). Briefly, the leaf samples were spray-inoculated with GZ8 (1 × 10^5^ mL^− 1^ spores) and submerged into 0.5 mg/ml NBT (Sigma). Then the stained leaves were incubated at 37 °C in the dark for 16 h and were de-stained with 95% alcohol at 65℃ to decolor the chlorophyll. The leaf sections were observed with a microscope (Zeiss Axio Imager A2).

DAB (Sigma Aldrich) was used as an indicator. In brief, leaves were collected and submerged into 0.5 mg/ml 3,3′-diaminobenzidine (DAB) solution and stained overnight. Next, the samples were de-stained with 95% alcohol at 65℃ to decolor the chlorophyll. Then the decolored samples were observed and pictured by a fluorescence microscope (Zeiss Axio Imager A2) or a stereomicroscope (OLYMPUS SZX16).

### Electronic Supplementary Material

Below is the link to the electronic supplementary material.

Supplementary Material 1

Supplementary Material 2

## Data Availability

All of the datasets are included within the article and its additional files.

## Abbreviations

PAMP: pathogen-associated molecular pattern
PTI: PAMP-triggered immunity
MAPK: Mitogen activated protein kinase
NOX: NADPH oxidase
RBOH: Respiratory burst oxidase homolog
ROS: Reactive oxygen species
DAB: 3,3’-diaminobenzidine
NBT: Nitroblue tetrazolium
RT-qPCR: Real-time quantitative polymerase chain reaction
LTH: Lijiang xin Tuan Heigu
IRBLkm-Ts: Pyricularia-Kanto51-m-Tsuyuake
TP309: Taipei309
CRISPR-Cas9: Clustered regularly interspaced short palindromic repeats-associated protein-9
