# Supplementary material for: Identification of NADPH Oxidase Genes Crucial for Rice Multiple Disease Resistance and Yield Traits
Source: Rice (N Y). 2024 Jan 3;17:1. doi: 10.1186/s12284-023-00678-5 (PMC10764683; doi:10.1186/s12284-023-00678-5)
Supplement: Supplementary file 1 — Supplementary Material 1 [file 12284_2023_678_MOESM1_ESM.pptx]

## Slide 1
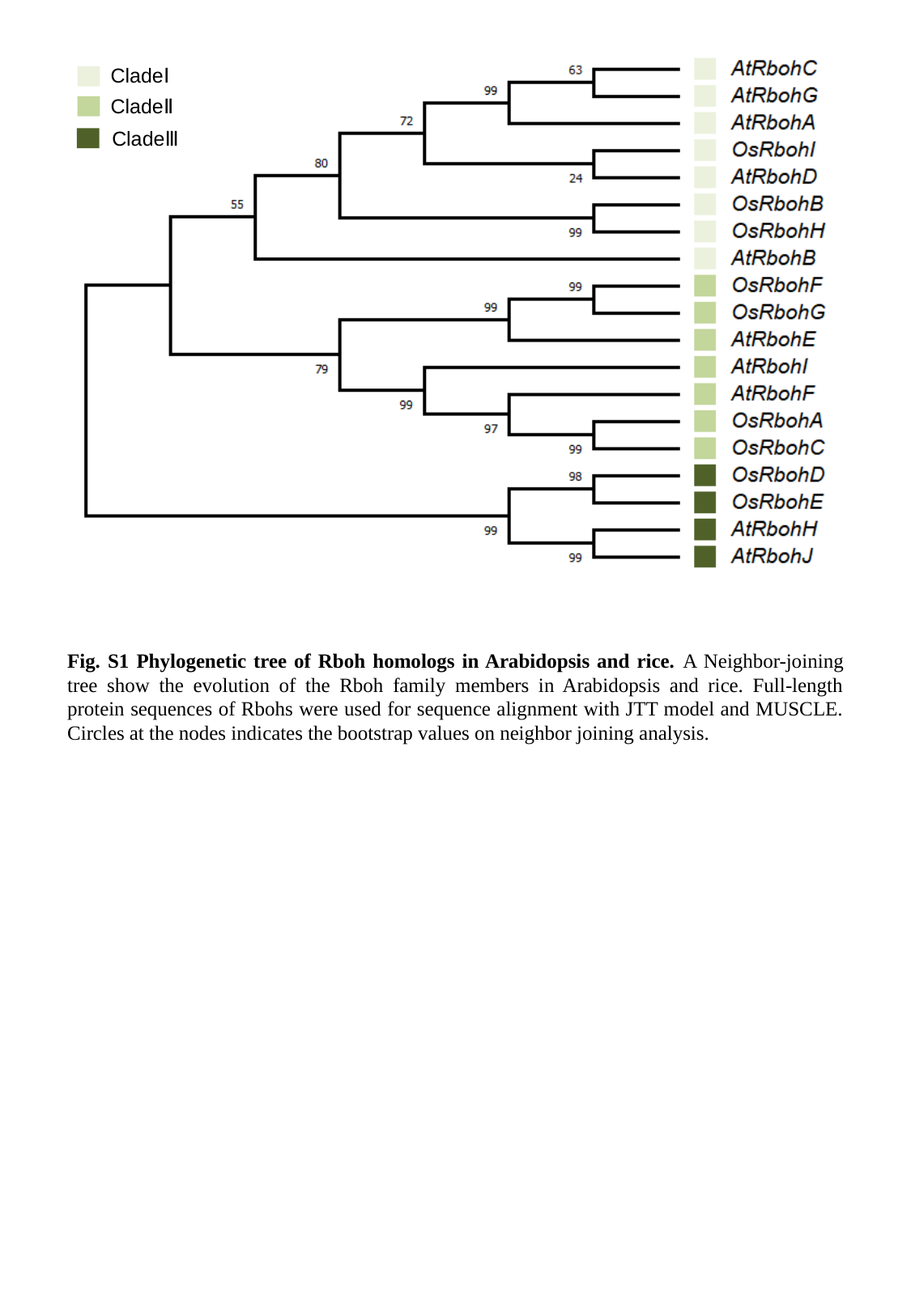

CladeⅠ
CladeⅡ
CladeⅢ
Fig. S1 Phylogenetic tree of Rboh homologs in Arabidopsis and rice. A Neighbor-joining tree show the evolution of the Rboh family members in Arabidopsis and rice. Full-length protein sequences of Rbohs were used for sequence alignment with JTT model and MUSCLE. Circles at the nodes indicates the bootstrap values on neighbor joining analysis.

## Slide 2
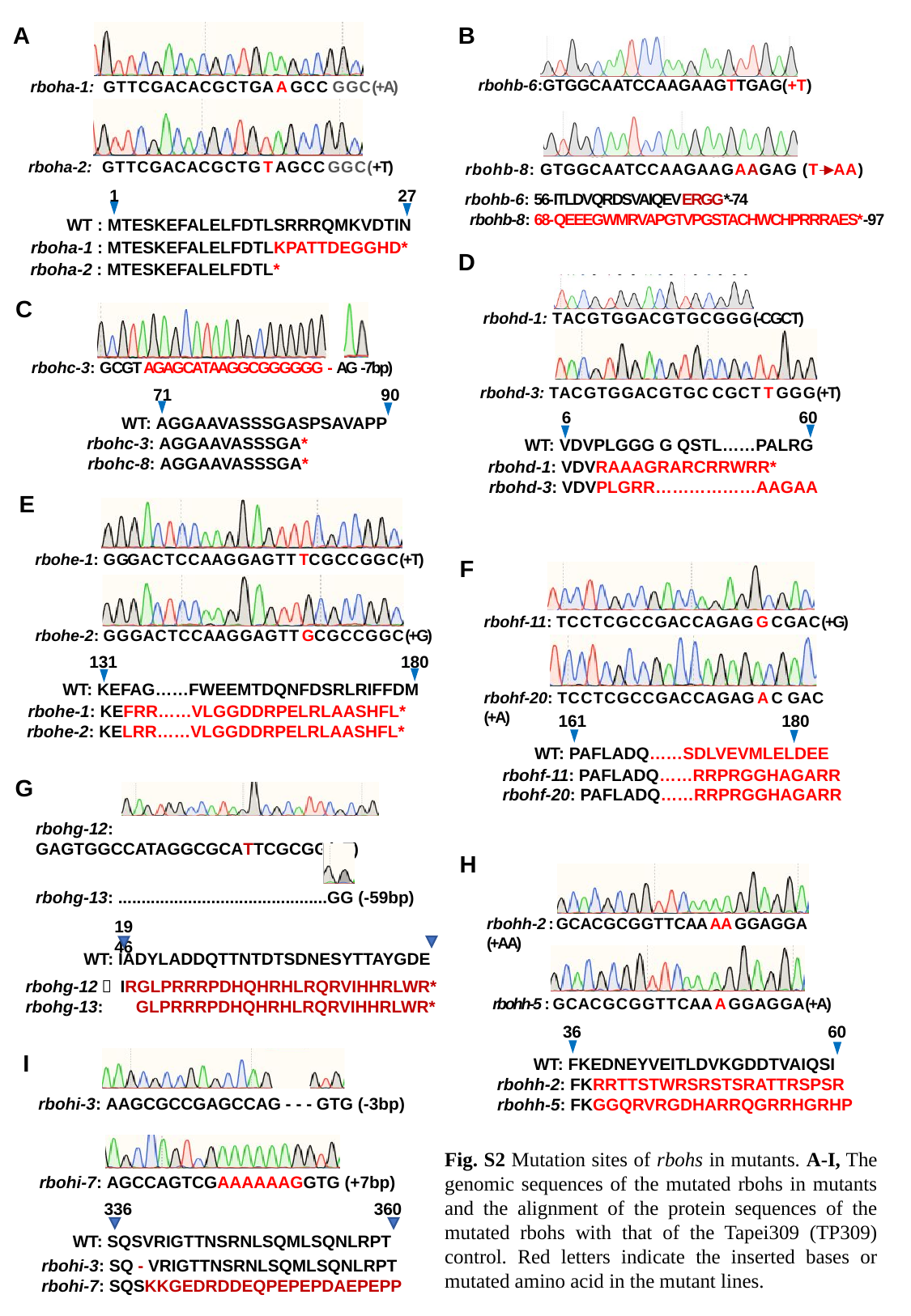

A
B
rboha-1: G T T C G A C A C G C T G A A G C C G G C (+A)
rboha-2: G T T C G A C A C G C T G T A G C C G G C (+T)
1 27
rboha-1 : MTESKEFALELFDTLKPATTDEGGHD*
rboha-2 : MTESKEFALELFDTL*
WT : MTESKEFALELFDTLSRRRQMKVDTIN
rbohb-6:GTGGCAATCCAAGAAGTTGAG(+T)
rbohb-8: GTGGCAATCCAAGAAGAAGAG (T AA)
rbohb-6: 56-ITLDVQRDSVAIQEVERGG*-74
rbohb-8: 68-QEEEGWMRVAPGTVPGSTACHWCHPRRRAES*-97
D
rbohd-1: T A C G T G G A C G T G C G G G (-CGCT)
rbohd-3: T A C G T G G A C G T G C C G C T T G G G (+T)
6 60
WT: VDVPLGGG G QSTL……PALRG
rbohd-1: VDVRAAAGRARCRRWRR*
rbohd-3: VDVPLGRR………………AAGAA
C
rbohc-3: G C G T A G A G C A T A A G G C G G G G G G - A G -7bp)
71 90
WT: AGGAAVASSSGASPSAVAPP
rbohc-3: AGGAAVASSSGA*
rbohc-8: AGGAAVASSSGA*
E
rbohe-1: G GG A C T C C A A G G A G T T TC G C C G G C (+T)
rbohe-2: G G G A C T C C A A G G A G T T GC G C C G G C (+G)
131 180
WT: KEFAG……FWEEMTDQNFDSRLRIFFDM
rbohe-1: KEFRR……VLGGDDRPELRLAASHFL*
rbohe-2: KELRR……VLGGDDRPELRLAASHFL*
F
rbohf-11: T C C T C G C C G A C C A G A G G C G A C (+G)
rbohf-20: T C C T C G C C G A C C A G A G A C G A C (+A)
161 180
rbohf-11: PAFLADQ……RRPRGGHAGARR
rbohf-20: PAFLADQ……RRPRGGHAGARR
WT: PAFLADQ……SDLVEVMLELDEE
G
rbohg-12: GAGTGGCCATAGGCGCATTCGCGG(+T)
rbohg-13: .............................................GG (-59bp)
19 46
WT: IADYLADDQTTNTDTSDNESYTTAYGDE
rbohg-12： IRGLPRRRPDHQHRHLRQRVIHHRLWR*
rbohg-13: GLPRRRPDHQHRHLRQRVIHHRLWR*
H
rbohh-2 : G C A C G C G G T T C A A A A G G A G G A (+AA)
rbohh-5 : G C A C G C G G T T C A A A G G A G G A (+A)
36 60
WT: FKEDNEYVEITLDVKGDDTVAIQSI
rbohh-2: FKRRTTSTWRSRSTSRATTRSPSR
rbohh-5: FKGGQRVRGDHARRQGRRHGRHP
I
rbohi-3: AAGCGCCGAGCCAG - - - GTG (-3bp)
rbohi-7: AGCCAGTCGAAAAAAGGTG (+7bp)
336 360
WT: SQSVRIGTTNSRNLSQMLSQNLRPT
rbohi-3: SQ - VRIGTTNSRNLSQMLSQNLRPT
rbohi-7: SQSKKGEDRDDEQPEPEPDAEPEPP
Fig. S2 Mutation sites of rbohs in mutants. A-I, The genomic sequences of the mutated rbohs in mutants and the alignment of the protein sequences of the mutated rbohs with that of the Tapei309 (TP309) control. Red letters indicate the inserted bases or mutated amino acid in the mutant lines.

## Slide 3
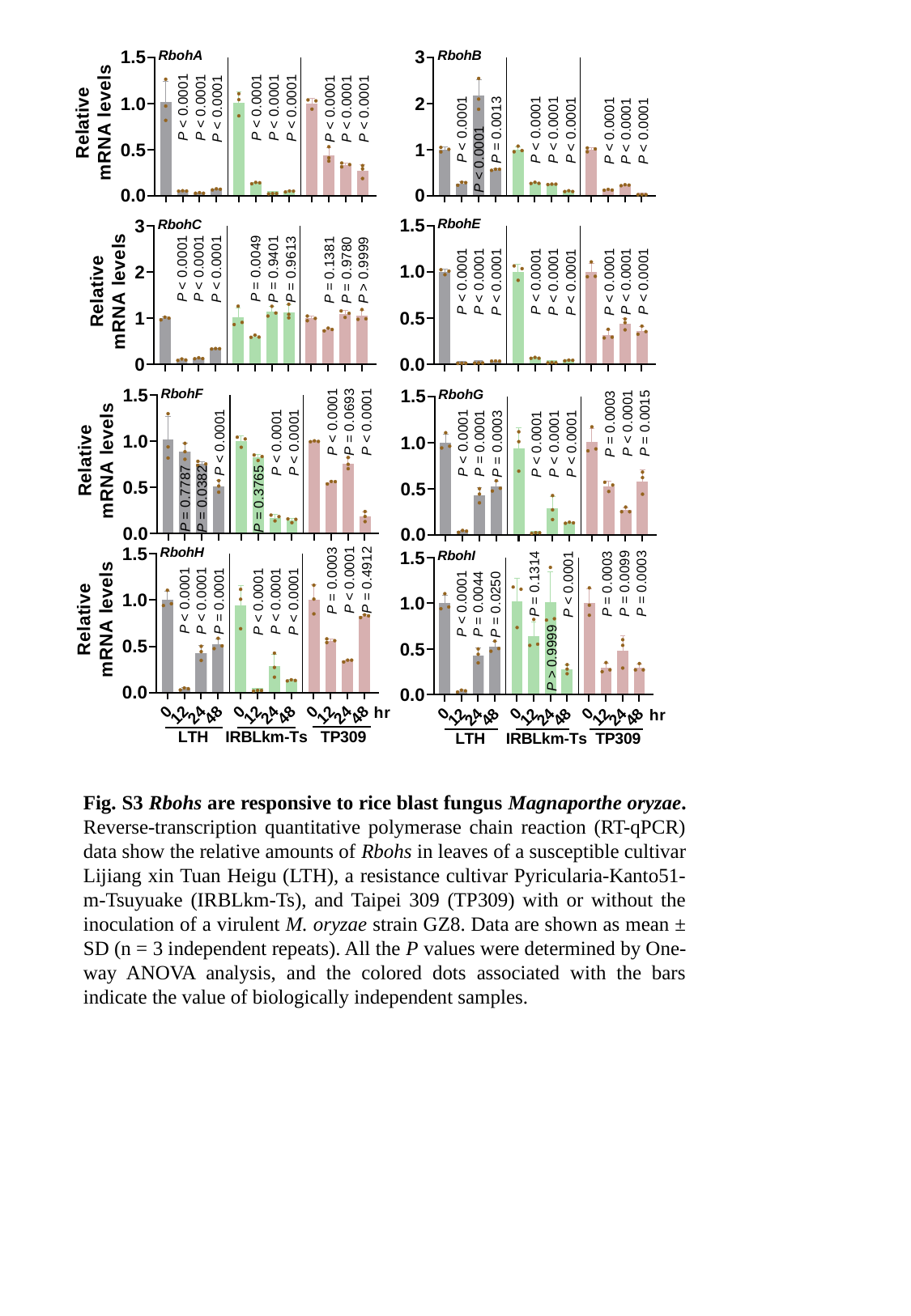

Fig. S3 Rbohs are responsive to rice blast fungus Magnaporthe oryzae. Reverse-transcription quantitative polymerase chain reaction (RT-qPCR) data show the relative amounts of Rbohs in leaves of a susceptible cultivar Lijiang xin Tuan Heigu (LTH), a resistance cultivar Pyricularia-Kanto51-m-Tsuyuake (IRBLkm-Ts), and Taipei 309 (TP309) with or without the inoculation of a virulent M. oryzae strain GZ8. Data are shown as mean ± SD (n = 3 independent repeats). All the P values were determined by One-way ANOVA analysis, and the colored dots associated with the bars indicate the value of biologically independent samples.
